# Morphology and taxonomic status of *Aedes aegypti* populations across Senegal

**DOI:** 10.1371/journal.pone.0242576

**Published:** 2020-11-18

**Authors:** Babacar Diouf, Ibrahima Dia, Ndeye Marie Sene, El Hadji Ndiaye, Mawlouth Diallo, Diawo Diallo

**Affiliations:** Pôle de zoologie médicale, Institut Pasteur de Dakar, Dakar, Sénégal; Universidade Nova de Lisboa Instituto de Higiene e Medicina Tropical, PORTUGAL

## Abstract

*Aedes aegypti* is the primary vector of dengue, Zika, yellow fever and chikungunya viruses to humans. In Africa, two subspecies, *Ae*. *aegypti aegypti* (*Aaa*) and *Ae*. *aegypti formosus* (*Aaf*) have been described. Until very recently, it was considered that the two forms were sympatric in East Africa and that only *Aaf* was present in Central and West Africa. However, recent data suggests that *Aaa* was also common in Senegal without any clear evidence of genetic differences with *Aaf*. This study was carried out in different *Ae*. *aegypti* populations from Senegal to better clarify their taxonomic status. The larvae, pupae and eggs were collected between July and September 2018 and reared individually to adult stage. For each population, F_1_ progeny from eggs laid by a single female F_0_ were reared as sibling samples. The number of pale scales on the first abdominal tergite (T_1_) and the basal part of the second tergite (T_2_) were counted. Individuals with no pale scale on T_1_ were classified as *Aaf* while those with at least one pale scale on this tergite were classified as *Aaa*. The morphological variations within families of *Aaf* were studied across 4 generations. In total, 2400 individuals constituting 240 families were identified, of which 42.5% were heterogeneous (families with both forms). Multivariate statistical analysis of variance including T_1_ and T_2_ data together showed that populations were significantly different from each other. Statistical analysis of T_1_ alone showed a similarity between populations from the southeast while variations were observed within northwest population. The analysis of family composition across generations showed the presence of *Aaa* and *Aaf* forms in each generation. The classification of *Ae*. *aegypti* into two subspecies is invalid in Senegal. Populations exhibit morphological polymorphism at the intra-family level that could have biological and epidemiological impacts.

## Introduction

Zika (ZIKV), dengue (DENV), yellow fever (YFV) and chikungunya (CHIKV) viruses are transmitted mainly by *Aedes aegypti* worldwide. These arboviruses have experienced a significant geographic expansion, causing epidemics in different countries of Africa, Indian Ocean, Asia, Pacific, Europe and America despite all the considerable efforts made for their control [1, 2]. DENV is the most prevalent arthropod-borne virus in the world. More than half of the world's population is exposed to dengue fever and the number of infections is estimated at 390 million each year [3]. The four serotypes (DENV1-4) have all been reported In Africa. Recently, epidemics of dengue 1 (DEN1) occurred in Senegal (2017 and 2018) and Burkina Faso (2019) [4]. A re-emergence of CHIKV has been observed [5] especially in Asia and Indian Ocean islands. ZIKV is the most frequently amplified arbovirus in Senegal [2, 6] and recently has risen to considerable notoriety worldwide [7]. Despite the availability of a highly effective vaccine YF outbreaks are still frequents in Africa [8], and has recently occurred in Democratic Republic of Congo and Angola from which imported cases have been reported in China [9, 10]. Forty-seven countries, including 34 in Africa and 13 in Central and South America, are endemic to YF [8, 11]. Without any specific treatment and licensed vaccines (with the exception of YF) against these arboviruses, vector control is the only way of effective prevention and control. However, this vector control requires very precise targeting of the populations actually involved in transmission and therefore better knowledge of their structuration. *Ae*. *aegypti*, the most important epidemic vectors of DENV, ZIKV, CHIKV, and YFV, is present in practically all tropical and intertropical areas especially between 35° North and 35° South latitudes [12]. It is genetically the best characterized species in the genus *Aedes* [13]. This species presents great morphological and behavioral variability, close proximity to humans and the ability to transmit many pathogens [14, 15]. The first observation of morphological variations in *Ae*. *aegypti* was made by Hill in 1921 in Queensland, Australia [16]. This author noted that populations of *Ae*. *aegypti* which bred in the bush was darker than that associated with urban environment. This implicit correlation between differences in color and behavior was among the many concerns that prompted Mattingly to reassess the biology and taxonomy of *Ae*. *aegypti* [14, 17]. Considering the morphological, ecological and ethological data, he described a pale anthropophilic form which breeds in urban environment and a dark or wild form preferring natural breeding sites and animals for blood meals. Following this correlation between the habitat, the morphology and the behavior of the females, this author conventionally subdivided the species into two subspecies: *Ae*. *aegypti aegypti* (*Aaa*) and *Ae*. *aegypti formosus* (*Aaf*). *Aaa* was considered as domestic and anthropophilic with at least one pale scale on the first abdominal tergite. As for, *Aaf* was described as darker and characterized by the total absence of pale scales on this first abdominal tergite. This form *Aaf* was supposed to be present only in Africa with sylvatic and rather zoophilic behaviors. Referring to Mattingly's work, McClelland proposed a classification of the different forms based one the coloration encountered, ranging from the black "F" form to the palest form "R" [15]. Applying this classification to a large population of *Ae*. *aegypti* from around the world, this author questioned the notion of subspecies as defined by Mattingly [14] and proposed the possibility of an incipient speciation [18]. Until very recently, it was considered that the two forms were sympatric in East Africa without reproductive barrier [19] and only *Aaf* would be present in Central and West Africa [14, 20]. However, recent data suggests that *Aaa* was also common in Senegal and presented a northwest–southeast cline with a dominance of *Aaa* in the northwest and *Aaf* in the southeast [21, 22]. Unlike morphological and bioecological data, the analysis of the genetic differentiation of *Ae*. *aegypti* populations from different localities in Senegal provides no clear evidence of the existence of two genetically distinct groups [21, 23]. In addition, genetic relationships highlighted by several molecular markers such as microsatellites and single nucleotide polymorphisms often do not match with morphological similarities [24]. Overall, these results improve understanding of the diversity of *Ae*. *aegypti* in West Africa, but so far, all these studies were done at population level and none has been interested in intra-family morphological variations. However, a study on the polymorphism within 196 families (each coming from a single female) from 18 anthropophilic and non-anthropophilic populations of *Ae*. *aegypti* collected from different localities in South Africa, showed that 60.2% of the families were heterogeneous containing both *Aaa* and *Aaf* individuals [25]. These results suggest that the classification of *Ae*. *aegypti* into two subspecies is not valid in South Africa. A similar study in West Africa could help to explain the lack of genetic structuring of *Ae aegypti* subspecies in this area. It is in this context that we conducted this study on intra-family morphological polymorphism in different populations of *Ae*. *aegypti* from Senegal to better clarify on their taxonomic status.

## Materials and methods

### Sampling sites

**Fig 1:**
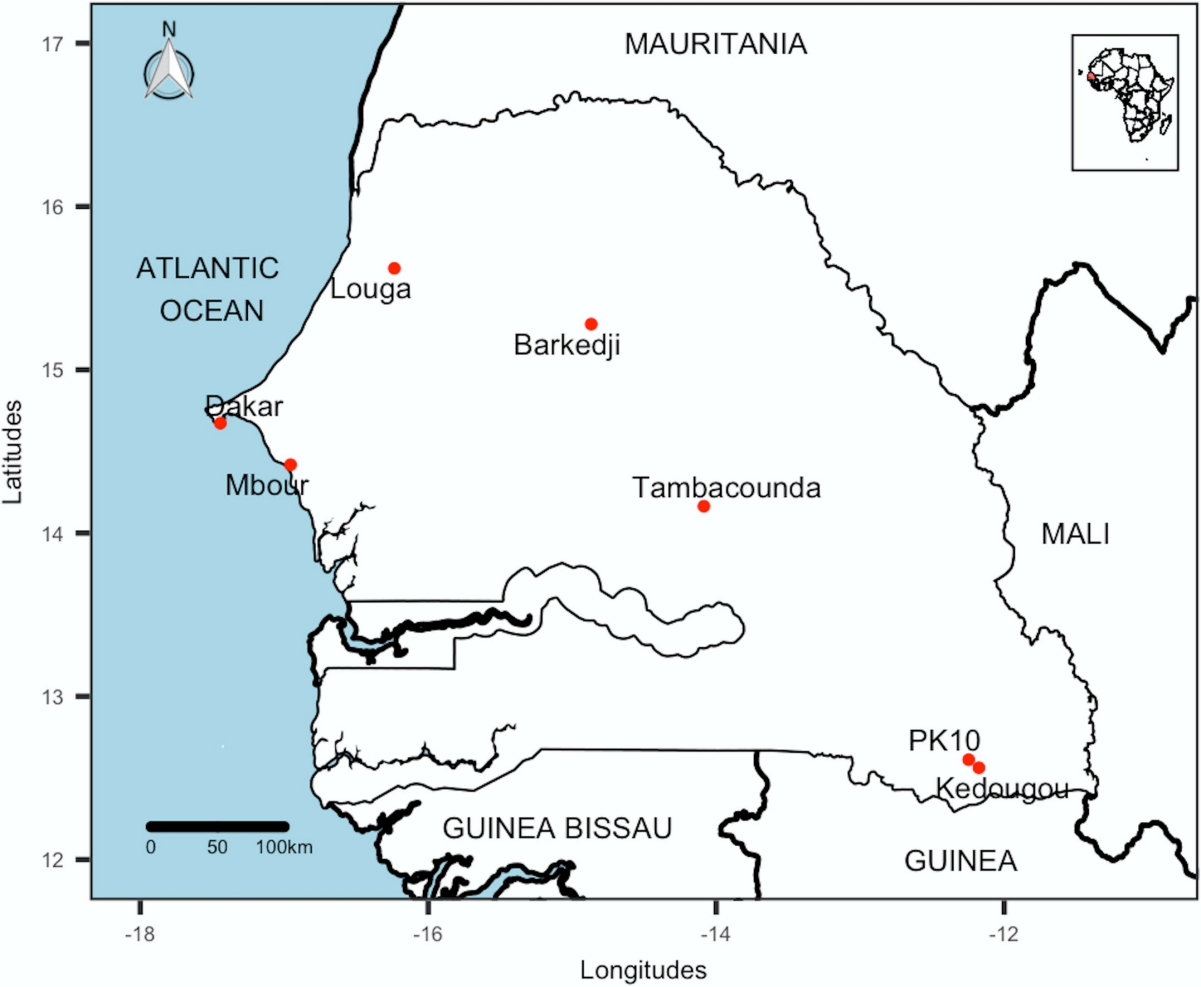
Collecting locations of *Ae*. *aegypti* populations, July-September 2018, Senegal. This map was created using the R software (version 4.0.2) and the package rgdal using an empty shapefile from the HDX website (https://data.humdata.org/dataset/senegal-administrative-boundaries) available under Creative Commons Attribution 4.0 International license.

Samples were collected between July and September 2018 in three climatic areas corresponding to three main rainfall area from south to north (Fig 1).

- Kédougou, PK10, and Tambacounda are located in the south of the country and are undergoing demographic and economic changes. They have a Sudano-Guinean and Sudano-Sahelian climate. They are a crossroads of ecosystems characterized by a very diverse flora and fauna which are the result partly of favorable climatic characteristics. It is the rainiest area in the country (between 450 and 1300 mm/year from May to October) with temperatures ranging from 21°C to 42°C.
- Dakar and Mbour are located in the west of the country dominated by a wooded savannah. They are among the most urbanized cities in Senegal. This area benefits from a coastal microclimate influenced by the trade winds and the monsoon. The relatively short hot and humid season lasts from July to October with mean temperatures around 27°C and annual rainfall of 300 to 400 mm/year.
- Barkédji and Louga located in the northwest of the country are characterized by a dry Sahelian climate with a vegetation consisting of a savannah with trees and a long dry season of 9 months or more. The short and unstable rainy season records rainfall between 300 mm and 390 mm in 24 rainy days. Temperatures range between 21°C to 38°C and relative humidity between 30 and 75% [26].

All sampling sites in Kédougou, Tambacounda, Dakar, Mbour, Barkédji, and Louga were located in the center of urban areas in the domestic environment while the sampling site at PK10 were within a forest gallery in a sylvatic environment.

### Field collection of samples

The samples were collected from various potential breeding sites of *Ae*. *aegypti* (Table 1). In the domestic environment, artificial breeding sites (used tires, bricks) were prospected to collect larvae and pupae. These immature stages were also collected in natural breeding sites (tree holes, fruit husks and rock holes) from one single forest gallery located at 10 km from Kédougou city (PK10). In this forest, the choice to study the variations of the two populations (PK10Aaf and PK10Aaa) was motivated by the first notification during our sampling of both forms (*Aaf* and *Aaa*) in sympatric in the natural breeding sites in contrast to previous data which only reported the presence of the *Aaf* form [21]. Eggs were collected with trap consisting of a black painted pot half filled with water in which an oblique piece of wood immersed at 2/3 was used as a laying substrate. These traps were hung in shaded places in the urban environment as well as in the forest. The samples were collected from sites at least 100 m apart.

**Table 1:** Breeding sites and collection stages of *Aedes aegypti* populations by locality, July-September 2018, Senegal.

*Locality*	*Latitude N*	*Longitude W*	*Breeding sites*	*Stages collected*	*Morphology of F_0_*
*Kédougou*	12°33’45.3”	12°10’31.9”	Used tires, bricks	Larvae + pupa	*Aaf*
*PK10 forest*	12°36’43”	12°14’46.80”	TH, RH, FH, Ovitraps	Larvae + pupa	*Aaa*, *Aaf*
*Tambacounda*	14°9’52.73”	14°5’8.98”	Used tires	Larvae + pupa	*Aaf*
*Louga*	15°37’16.5”	16°14’7.6”	Ovitraps	Eggs	*Aaa*
*Barkédji*	15°16’37.4”	15°51’46.8”	Ovitraps	Eggs	*Aaa*
*Mbour*	14°25’7.6”	16°57’23.1”	Used tires, poultry waterers	Larvae + pupa	*Aaa*
*Dakar*	14°40’22.5”	17°26’36.9”	Used tires, flower pots	Larvae + pupa	*Aaa*

TH = Tree holes, RH = Rock holes, FH = Fruit husks.

### Ethics statement

No specific permits were required for this study. No specific permissions were required for these activities and the locations investigated are not protected. This study did not involve endangered or protected species. The study protocol was carefully explained to the heads and inhabitants of each household investigated to obtain their informed oral consent.

### Sample processing in the laboratory

Larvae and eggs were maintained under standard insectarium conditions [27] (temperature of 27± 1° C, relative humidity of 80% and a photoperiod of 12: 12h) until pupal stage. These pupae, as well as those collected directly in the field, were individually placed in test tubes.

After their emergence, adult mosquitoes (F_0_) were identified morphologically according to the descriptions of Mattingly and Huang [14, 28] and then grouped by sex. Subsequently, they were gently cold-anesthetized and their wings were spread using needles to check the presence or not of pale scales on the first abdominal tergite under binocular dissecting microscope. After identification, male and female individuals of the same form were pooled according to their origin for mating. The *Aaa* forms were chosen in Dakar, Mbour, Barkedji and Louga for the F_0_ parents while *Aaf* was chosen in Tambacounda and Kédougou and both forms in PK10 forest (Table 1).

### F_0_ families egg batches production

For each population, 30 fully engorged *Aaa* or *Aaf* females were selected for individual egg batches production. Each female was placed in a cup covered with a mosquito net and a cotton wool soaked in water were deposited at the bottom to collect the eggs. These females were subsequently fed with 10% sucrose and maintained under standard insectarium conditions as describe previously.

### Morphology of F_1_ progeny

Each egg batch (family) was reared separately into adults, and 10 F_1_ females identified. For that, the wings of the mosquitoes were cut off at their base and theirs bodies fixed using a needle horizontally crossing the thorax. The number of pale scales on the first abdominal tergite (T_1_) and the basal part of the second tergite (T_2_) were counted under a binocular dissecting microscope (Motic ST-36C-6LED) at 40 times magnification.

To follow the morphological variations within families across 4 generations, egg batches were produced from 5 pairs of *Aaf* for each generation. The different egg batches were separately reared into adults and 10 females identified, as described previously, per egg batch.

### Data analysis

Mosquito specimens without any pale scale on the T_1_ tergite were classified *Aaf* while those with one or more pale scales on this T_1_ tergite were classified *Aaa*. Thus, families in which all individuals had the same forms (*Aaa* or *Aaf*) were considered as homogeneous while families which presented both forms (*Aaa* and *Aaf*) were considered as heterogeneous. The mean numbers of pale scales on both tergites (T_1_ + T_2_) of the different populations were compared by multivariate analysis of variances using the Wilk’Lambda test. The mean numbers of pale scales on each tergite (T_1_ and T_2_) were compared using the Waller-Duncan t-test [29]. The relative abundance of the two forms across the generations was compared with the χ^2^ test. Statistical analyses were performed using the R software version 2.15.1 [30] and results were considered significant when P < 0.05.

## Results

**Fig 2:**
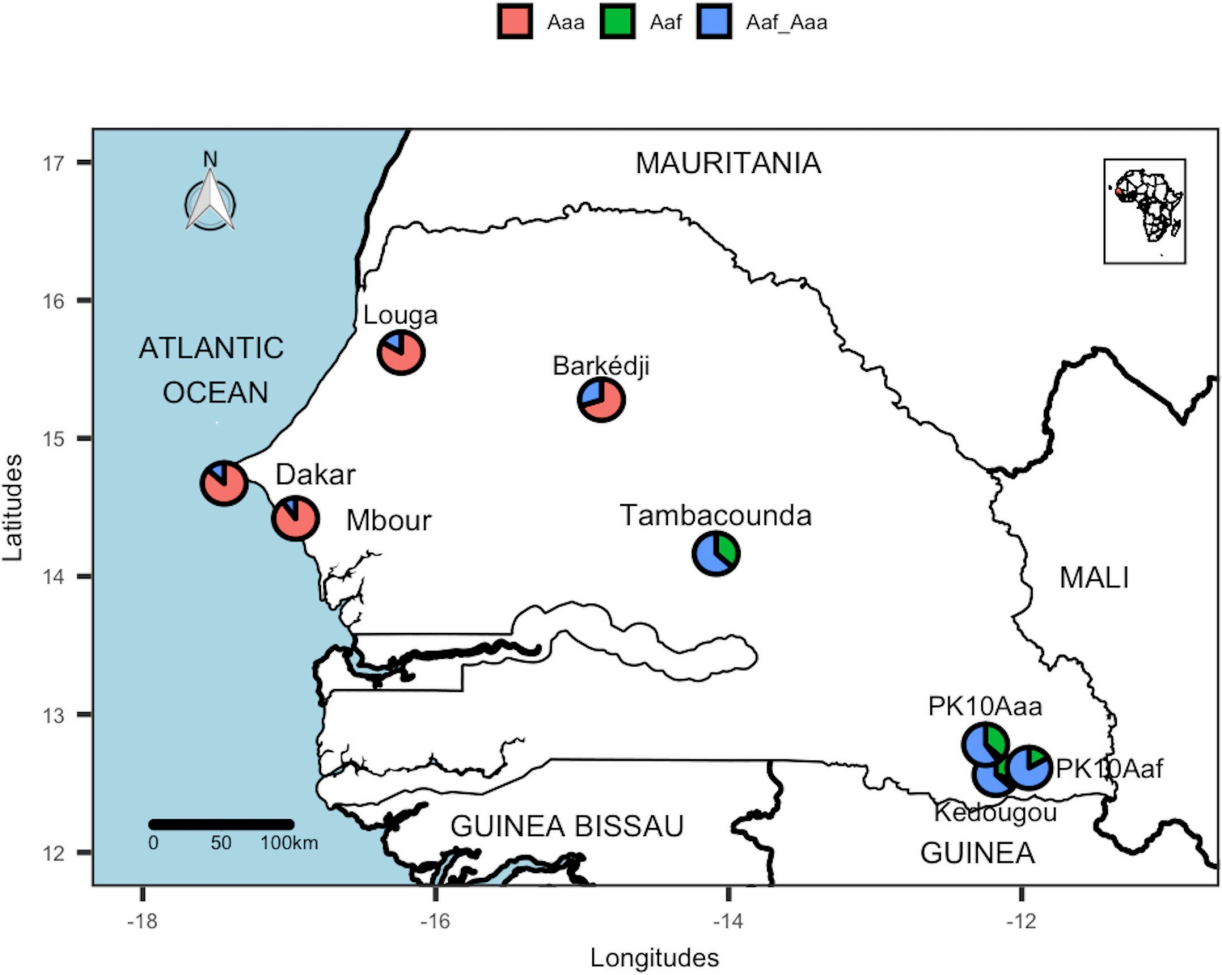
Proportion of homogeneous *Aaa*, homogeneous *Aaf* and heterogeneous (*Aaf* + *Aaa*) families of *Ae*. *aegypti* populations, July-September 2018, Senegal. Two populations of *Ae*. *aegypti* from the *Aaa* (PK10Aaa) and *Aaf* (PK10Aaf) parents were investigated in the PK10 forest Site. This map was created using the R software (version 4.0.2) and the package rgdal using an empty shapefile from the HDX website (https://data.humdata.org/dataset/senegal-administrative-boundaries) available under Creative Commons Attribution 4.0 International license.

A total of 2400 female progenies belonging to 240 families were identified and the number of pale scales on their T_1_ and T_2_ counted. Out of the 240 families, 42.5% (102/240) and 15% (36/240) were respectively *Aaa* and *Aaf* homogeneous families. The remaining families (42.5% of the 240 families investigated) were heterogeneous, containing both the *Aaa* and *Aaf* forms (Table 2). For each population studied, part of the F_1_ offspring were morphologically different from their F_0_ parents. Populations from the southeast (Kédougou, PK10 and Tambacounda) presented higher heterogeneity rates (Fig 2) compared to those from the northwest (Dakar, Mbour, Louga and Barkédji) (P <0.05).

**Table 2:** Comparison of the average numbers of pale scales on T_1_ and T_2_ tergites and classification of F_1_ families in 8 *Ae*. *aegypti* populations, July-September 2018, Senegal.

		T_1_				T_2_				Number of families		
Locality	Nb	min-max	Mean	sd	p-value	min-max	Mean	sd	p-value	*Aaa*	*Aaf*	*Aaf*+*Aaa*
Barkédji	300	0–122	40.09	22.04	a	0–68	17.62	12.76	a	21	0	9
Dakar	300	0–83	25.77	16.31	b	0–76	8.13	10.13	b	26	0	4
Louga	300	0–124	31.76	18.18	c	0–42	16.23	10.43	a	25	0	5
Mbour	300	0–87	31.89	14.45	c	0–04	12.00	8.49	c	27	0	3
PK10Aa	300	0–35	2.36	5.03	d	0–17	1.56	2.91	df	1	11	18
Kédougou	300	0–23	2.66	4.72	d	0–23	0.92	2.56	d	2	9	19
PK10Af	300	0–32	1.79	4.37	d	0–16	6.89	2.77	eb	0	5	25
Tambacounda	300	0–34	2.39	6.04	d	0–23	3.35	5.18	f	0	11	19
*Total*	2400									102	36	102
										42.50%	15%	42.50%

Identical letters indicate populations with a comparable average number of scales. Nb, number of individuals examined; sd, standard deviation; min, minimum number of scales; max, maximum number of scales; *Aaa*, homogeneous family *Ae*. *aegypti aegypti*; *Aaf*, homogeneous family *Ae*. *aegypti formosus*; *Aaf* + *Aaa*, heterogeneous family.

The Waller-Duncan t-test on T_1_ showed a similarity between *Ae*. *aegypti* populations from Kédougou, PK10 and Tambacounda (Table 2) with an average number of 1 to 2 pale scales on T_1_ (p> 0.05). However, variations were noted within *Ae*. *aegypti* populations from northwest sites with on average of pales scales ranging from 31 to 40. The population from Barkédji showed more pale scales on T_1_ (p <0.0001) whereas that from Dakar presented fewer pale scales on T_1_ than the others (p <0.0001). On the other hand, the populations from Mbour and Louga were comparable (p> 0.05). All of these populations had more pale scales on T_1_ than those from the southeast (p <0.0001).

The same analysis made on T_2_ showed significant variations between populations from the southeast and those from the northwest with the exception of Dakar and PK10Aaf which were comparable (Table 2). A similarity was noted between populations from Barkédji and Louga (p > 0.05) and between those from Kédougou and Tambacounda (p > 0.05).

When both tergites means (T_1_ + T_2_) were compared together by multivariate analysis, significant variations were observed across the 8 populations studied (p < 0.000 1).

**Fig 3:**
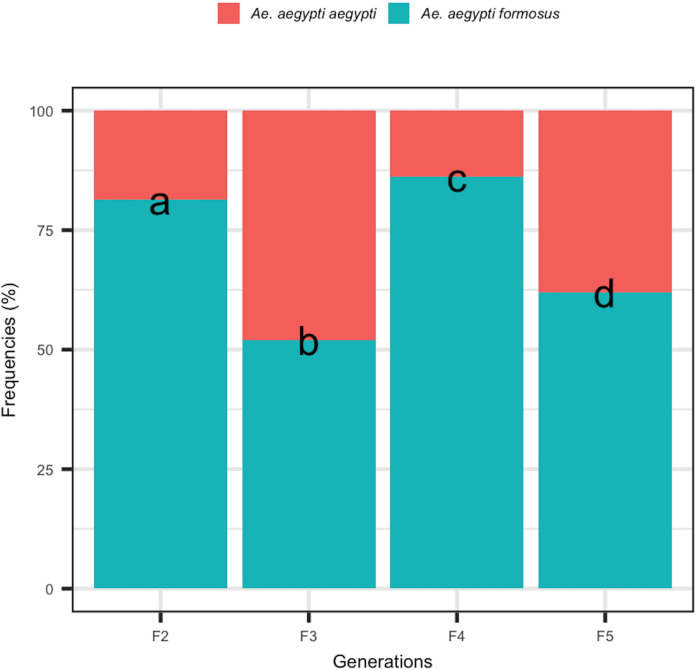
Relative abundance of *Aaa* and *Aaf* forms across four generations (F_2_ to F_5_) of *Aaf* parents. Different letters indicate a significant difference from one generation to another.

The analysis of the composition of the offspring of *Aaf* families across 4 generations showed that the two forms (*Aaa* and *Aaf*) were present in each generation (Fig 3). The relative abundance of the two forms was statistically different from one generation to another (p <0.05).

## Discussion

This study conducted on 2400 females belonging to 240 families, showed significant intra-family morphological variations in different populations of *Ae*. *aegypti* from Senegal. Our data showed that a part of the F_1_ progeny was morphologically different from their F_0_ parents whatever the population studied. This intra-family heterogeneity in the offspring noted during this study suggest that the classification of *Ae*. *aegypti* into two subspecies by Mattingly [14] based on the presence of pale scales on the first abdominal tergite (T_1_) should be considered as invalid in Senegal. The presence of both forms (*Aaf* and *Aaa*) across the country (at family level) is discordant with an earlier study which showed a southeast/northwest cline in the distribution of these two forms at population level with the exclusive presence of *Aaf* in the southeast, *Aaa* in the northwest and both forms in the center of the country [21]. The non-detection of *Aaa* in the southeast and *Aaf* in the northeast, in this previous study, could be explained by their low intra-family proportions in the populations studied and the possible influence of some factors (temperature, relative humidity, rainfall, etc.) favoring a high mortality of the less represented forms. The impact of these factors could explain the southeast/northwest cline of *Aaa* and *Aaf* as previously observed [21]. Comparative studies on the survival of both forms from the southeast, center and northeast of Senegal are necessary to assess the possible impact of these factors. Our results are similar to those obtained during a study of the polymorphism of *Ae*. *aegypti* populations in South Africa [25]. Indeed, the author by rearing separately the progenies of several females of *Aaf* or *Aaa* has observed the presence of both forms in several families. As in South Africa, the proportion of homogeneous *Aaf* families was lower than that of the homogeneous *Aaa* and the heterogeneous families. However, the proportion of homogeneous *Aaf* families was more important in Senegal. These intra-family morphological variations could explain the significant variations observed within and between different *Ae*. *aegypti* populations worldwide. These variations also explain the presence in sympatry of both forms, formerly considered as 2 different subspecies, in sub-Saharan Africa from natural and artificial breeding sites [14, 21, 31–33]. It is interesting to note that our results are in perfect agreement with all the genetic studies which did not show any clear differentiation between individuals belonging to the two forms collected in several localities of Senegal [21–23, 34, 35]. The intra-family morphological variations of *Ae*. *aegypti* populations in other parts of Africa should be systematically reviewed to determine their taxonomic status. The heterogeneity rates in the progeny were higher in the populations from the southeast (Kédougou, PK10 and Tambacounda) compared to those from the northwest (Dakar, Mbour, Louga and Barkédji). That could be explained by an increase of *Aaa* specimens in this area in correlation with the beginning of an adaptation to the domestic environment of these so-called wild populations. Consistent with these data, a recent study showed the existence of a highly anthropophilic neo-population of *Ae*. *aegypti* in this area (unpublished data). Based on the average number of pale scales on T_1_ tergite, our results showed a similarity in populations from the southeast (Kédougou, PK10 and Tambacounda). This could be explained by a conservation of sylvatic characters, in particular the dark coloring of tergites in these populations evolving under the same ecological conditions and dominated by the *Aaf* form [21, 23]. Indeed, an impact of climatic factors including temperature and relative humidity on the geographic distribution of the two forms has been noted by some authors [32, 36]. In agreement with other studies, our results showed variations in coloration on the basal part of T_2_ ranging from complete absence to well-marked bands [15, 25]. These variations less reflected the geographic distribution of the two forms in Senegal compared to T_1_.

The presence of both forms across the four generations of *Aaf* parents seems to confirm that the coloring of scales on T_1_ is a polymorphic morphological character within families. *Ae*. *aegypti* should be viewed as a highly polymorphic rather than a polytypic species.

Other studies reported chromosomal inversions in *Aaf* from Senegal [37] and elsewhere with genetic introgression between *Aaa* and *Aaf* [38] suggesting a chromosomal polymorphism in *Ae*. *aegypti* populations. These chromosomal inversions have been directly associated with behaviors such as feeding behavior [39, 40], oviposition site preferences [41, 42], insecticide resistance [43] and immune response to parasites [44, 45] in malaria mosquito vectors. Moreover, mutant markers for abdominal coloring have been reported on *Ae*. *aegypti* chromosomes [13, 46].

Many studies showed that *Aaa* populations have higher vector competence compared to *Aaf* [21, 47]. This difference in vector competence could be linked to differences in competence of individuals of both forms. Thus, to test this hypothesis, it would be interesting to compare the intra-family variations in vector competence of individuals belonging to the *Aaf* and *Aaa* forms.

## Conclusion

This study revealed a morphological polymorphism at intra-family level in different populations of *Ae*. *aegypti* from Senegal. The presence of pale scales on T_1_ as a classification criterion for the two forms should be considered as invalid in Senegal. However, this study reveals two distinct groups; a group located in the southeast of the country with an average of 1 to 2 pale scales on T_1_ and another group in the northwest with an average of 31 to 40 pale scales on same tergite. Additional detailed chromosome and/or genomic studies could give more explanation to these intra-family and inter-population morphological variations which could have an impact on biological parameters and the transmission of pathogens by *Ae*. *aegypti* in Senegal.
